# Does meniscal tear morphology independently predict repair failure? A context‐adjusted analysis of 500 consecutive arthroscopic meniscal repairs

**DOI:** 10.1002/jeo2.70947

**Published:** 2026-10-09

**Authors:** Horacio Rivarola, Cristian Collazo, Marcos Palanconi, Marcos Meninato, Carlos Peñaherrera‐Carrillo, Alejandro Barros Castro, Francisco Endara Urresta

**Affiliations:** ^1^ Hospital Universitario Austral Buenos Aires Argentina; ^2^ Hospital Vozandes Quito Ecuador; ^3^ Centriqo Quito Ecuador; ^4^ Clínica Arthros Quito Ecuador

**Keywords:** arthroscopic meniscal repair, Cox regression, meniscal preservation, meniscal repair, meniscal tear morphology, prognostic model, repair failure, survival analysis

## Abstract

**Purpose:**

To determine whether meniscal tear morphology independently predicts repair failure following arthroscopic meniscal repair after adjustment for patient‐, meniscal‐ and repair‐related characteristics and to quantify the incremental prognostic value of tear morphology beyond established clinical predictors.

**Methods:**

A retrospective multicenter cohort study included 500 consecutive patients undergoing primary arthroscopic meniscal repair with a minimum follow‐up of 24 months. Repairable tear morphologies included longitudinal vertical, bucket‐handle, radial, horizontal cleavage, complex, ramp and root tears. Patients undergoing revision meniscal repair, previous ipsilateral meniscal repair or transplantation, treatment of discoid meniscus, repair of degenerative root tears associated with advanced osteoarthritis, concomitant corrective osteotomy or arthroplasty or with insufficient operative or follow‐up data were excluded. Meniscal characteristics included tear morphology, laterality, vascular zone and intraoperatively assessed tissue quality based on structural integrity, macroscopic degeneration and suture‐holding capacity. Repair‐related variables included repair technique (all‐inside, inside‐out or hybrid), number of fixation sutures, suture density and concomitant anterior cruciate ligament reconstruction. Repair failure was defined as subsequent ipsilateral meniscal reoperation. Kaplan–Meier analysis and multivariable Cox proportional hazards regression were used to evaluate repair survival and independent predictors of failure.

**Results:**

During a median follow‐up of 45 months, 81 patients (16.2%) underwent reoperation. Five‐year repair survivorship was 82.6%. Although repair survival differed among tear morphologies in univariable analysis (log‐rank *p* = 0.012), tear morphology was no longer independently associated with failure after multivariable adjustment. Independent predictors of failure included medial meniscal repair (hazard ratio [HR] 2.18), poor tissue quality (HR 2.74), avascular tear location (HR 1.89), lower suture density (HR 0.71 per additional suture/cm) and absence of concomitant anterior cruciate ligament reconstruction (HR 1.67). Addition of tear morphology to the clinical prediction model produced only a modest improvement in discrimination (Harrell's C‐index 0.782 vs. 0.794) and minimal improvement in calibration.

**Conclusion:**

Meniscal tear morphology demonstrated limited independent prognostic value after adjustment for patient‐, lesion‐ and procedure‐related characteristics. Prognosis after arthroscopic meniscal repair appears to depend more strongly on the biological and mechanical context of the reparable meniscus than on tear configuration alone. Clinical decision‐making should therefore move beyond descriptive tear classification toward comprehensive contextual assessment integrating tissue quality, vascularity, meniscal laterality and repair‐related factors.

**Level of Evidence:**

Level III.

**Abbreviations**
AIC: Akaike information criterion
BMI: body mass index
CI: confidence interval
HR: hazard ratio
IQR: interquartile range
MRI: magnetic resonance imaging
ROC: receiver operating characteristic
STROBE: Strengthening the Reporting of Observational Studies in Epidemiology

## INTRODUCTION

Meniscal preservation has become a fundamental principle of contemporary knee surgery because of the meniscus' essential role in load transmission, shock absorption, joint stability, lubrication and long‐term chondral protection. Arthroscopic meniscal repair has therefore progressively replaced partial meniscectomy whenever technically feasible, resulting in improved functional outcomes and a lower risk of subsequent osteoarthritis. Nevertheless, clinical success remains inconsistent, with systematic reviews reporting failure or revision rates approaching 20% at mid‐ to long‐term follow‐up, with substantial variability among studies according to patient selection, tear characteristics, concomitant procedures, outcome definition and duration of follow‐up [1, 12, 13, 25, 26, 27, 34, 35]. This substantial variability suggests that successful healing depends on a complex interaction of patient‐, lesion‐ and procedure‐related factors rather than on surgical repair alone. Although numerous predictors of repair failure have been proposed, their relative importance remains incompletely understood, limiting accurate preoperative risk stratification and individualized surgical decision‐making [7, 8, 26, 27, 32, 34, 35, 38, 39].

Among meniscal characteristics, tear morphology continues to play a central role in clinical decision‐making. Repair indications, surgical technique, fixation strategy and postoperative rehabilitation are frequently influenced by whether a tear is classified as longitudinal, bucket‐handle, radial, horizontal cleavage, complex or root. This morphology‐based approach is clinically intuitive because different tear configurations represent distinct mechanical disruptions and may require different repair strategies.

However, reported outcomes vary among tear configurations. Contemporary studies indicate that complex, radial and posterior root tears may experience clinically relevant failure rates, whereas more favourable outcomes are frequently reported for appropriately selected simple longitudinal tears [1, 12, 13, 22, 23, 25, 34]. Medial meniscus posterior root repair has recently been associated with a 22.7% rate of clinical or radiological failure, while contemporary studies of radial and complex tear repair similarly demonstrate that failure remains clinically relevant despite generally favourable clinical outcomes [12, 13, 25]. In paediatric and adolescent populations, outcomes have also been shown to vary according to tear type and location [1].

Importantly, tear morphology is not biologically independent from other lesion characteristics. Different tear configurations arise within different clinical and tissue environments. Horizontal cleavage tears and medial meniscus posterior root tears are more frequently associated with degenerative tissue and chondral abnormalities, whereas vertical longitudinal tears are more commonly associated with traumatic mechanisms, particularly in younger and ligament‐injured knees. Consequently, an apparent association between morphology and repair failure may partly reflect differences in tissue quality, vascularity, chronicity, cartilage status and patient phenotype rather than an independent effect of geometric tear configuration itself.

These relationships may explain why the available literature remains inconsistent regarding the independent prognostic importance of tear morphology. Some investigations have identified morphology‐dependent differences in repair survival, whereas others have demonstrated attenuation of these associations after adjustment for demographic, biological and surgical factors [1, 7, 8, 12, 13, 25, 26, 27, 32, 34, 35, 38, 39]. Thus, tear morphology may be better interpreted as one component of a broader biological and mechanical context rather than as an isolated determinant of healing.

Importantly, tear morphology is not biologically independent from other lesion characteristics. Different tear configurations arise within different clinical and tissue environments: horizontal cleavage tears and medial meniscus posterior root tears are more frequently encountered in menisci with degenerative changes and associated chondral pathology, whereas vertical longitudinal tears are more commonly associated with traumatic mechanisms, particularly in younger and ligament‐injured knees. Consequently, an apparent association between tear morphology and repair failure may partly reflect differences in underlying tissue quality, vascularity, chronicity, cartilage status and patient phenotype rather than an effect of geometric tear configuration alone [1, 12, 13, 25].

The clinical significance of a given tear configuration is likely modified by meniscal laterality, anterior cruciate ligament status, tissue quality, vascular zone involvement, chronicity, repair construct and associated intra‐articular pathology. Supporting this concept, a recent multicenter study including 1008 isolated meniscal repairs demonstrated that medial meniscal repair, rather than tear morphology itself, independently predicted failure after multivariable adjustment [8]. Although this study represents the largest contemporary analysis of isolated repairs, several clinically relevant variables—including detailed tissue quality, lesion chronicity, repair construct characteristics and the incremental prognostic contribution of morphology beyond established clinical factors—were not comprehensively evaluated. Consequently, whether tear morphology independently contributes meaningful prognostic information after accounting for the broader clinical context remains uncertain.

The purpose of this study was to determine whether meniscal tear morphology independently predicts failure following arthroscopic meniscal repair after adjustment for patient‐, lesion‐ and repair‐related variables and to quantify the incremental prognostic value of tear morphology beyond established clinical predictors.

It was hypothesized that tear morphology would demonstrate a significant univariable association with repair failure but that its independent prognostic effect would be substantially attenuated after comprehensive multivariable adjustment.

## METHODS

### Study design

A retrospective multicenter cohort study was conducted in accordance with the Strengthening the Reporting of Observational Studies in Epidemiology (STROBE) statement and was designed following the methodological principles recommended for prognostic research. Consecutive patients who underwent arthroscopic meniscal repair at two tertiary referral sports medicine centres were identified from prospectively maintained institutional surgical databases. Institutional Review Board approval was obtained at both participating institutions before data collection, and all procedures complied with the ethical principles outlined in the Declaration of Helsinki. Because of the retrospective design and anonymized data collection, informed consent was waived according to local institutional regulations.

### Patient selection

All consecutive patients who underwent primary arthroscopic meniscal repair during the study period were screened for eligibility (Figure 1). Eligible patients were required to have undergone primary arthroscopic repair of a meniscal tear considered repairable according to intraoperative assessment, with complete operative documentation, preoperative magnetic resonance imaging (MRI) available for review and a minimum clinical follow‐up of 24 months or documented repair failure before completion of the minimum follow‐up period.

**Figure 1:**
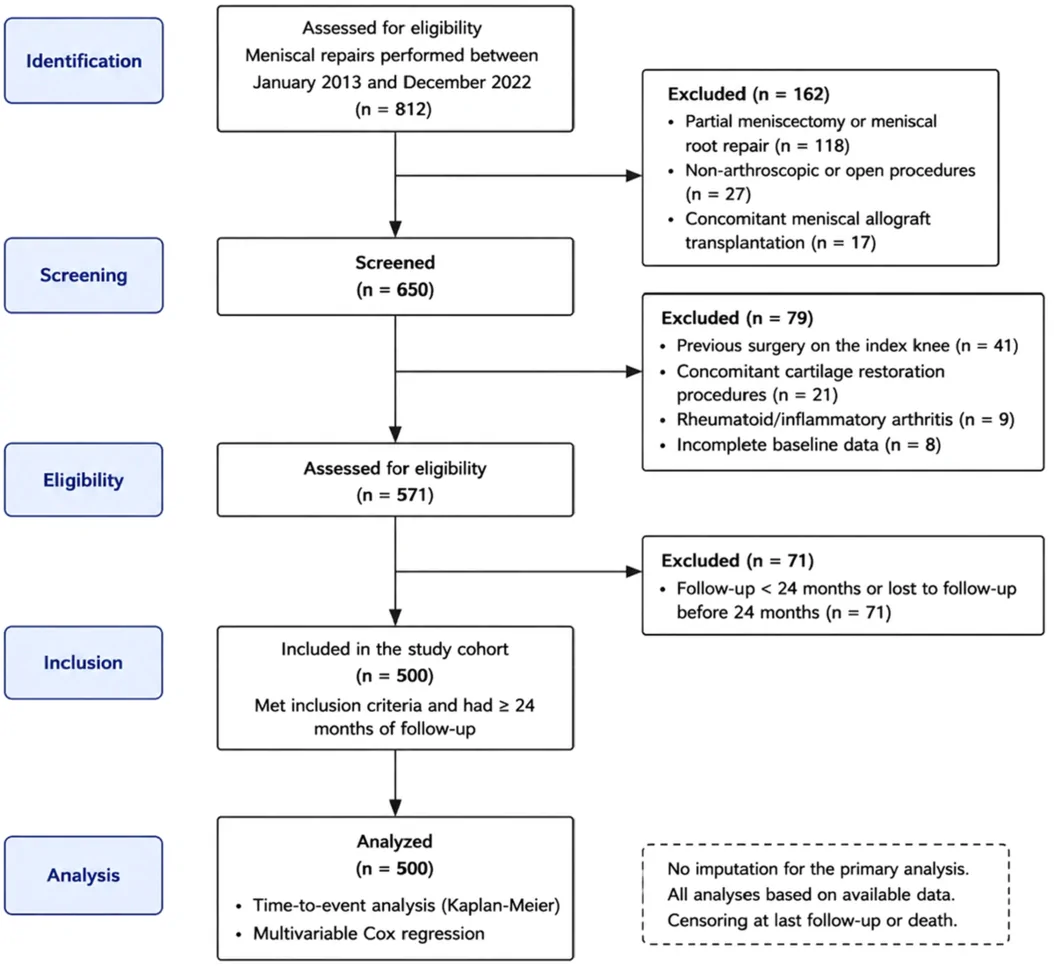
STROBE flow diagram. Flow diagram illustrating patient selection according to STROBE recommendations. The diagram summarizes the screening process, eligibility assessment, exclusion criteria and final inclusion of 500 consecutive patients who underwent arthroscopic meniscal repair and completed the minimum follow‐up required for survival analysis. STROBE, Strengthening the Reporting of Observational Studies in Epidemiology.

Repairable meniscal root tears without advanced degenerative joint changes were eligible for inclusion. Root tears associated with Kellgren–Lawrence grade ≥3 osteoarthritis were excluded. Patients with missing follow‐up data, unavailable operative reports or insufficient information regarding the primary outcome were also excluded. A STROBE flow diagram summarizes patient identification, exclusions and the final study cohort.

### Surgical technique

All procedures were performed arthroscopically by fellowship‐trained knee surgeons using contemporary repair techniques according to individual lesion characteristics and surgeon preference. Meniscal repair techniques were classified as all‐inside, inside‐out or hybrid repair. Hybrid repair was defined as the use of more than one repair technique within the same meniscal repair. The decision to preserve and repair the meniscus was based on intraoperative assessment of tear reducibility, residual tissue quality, vascularity, mechanical stability after reduction and anticipated healing potential rather than tear morphology alone. Lesions considered irreparable because of severe tissue degeneration, inability to obtain stable reduction or inadequate remaining meniscal substance underwent alternative treatment and were not included in the present analysis.

### Variables collected

Demographic, clinical, imaging, operative and follow‐up variables were extracted from prospectively maintained electronic medical records and operative reports using standardized data collection forms.

Patient‐related variables included age at surgery, sex, body mass index (BMI), smoking status, preinjury participation in pivoting sports, competitive athletic participation, interval from injury to surgery and concomitant anterior cruciate ligament reconstruction performed at the index procedure.

Lesion‐related variables included meniscal laterality, involved meniscal segment, tear morphology, vascular zone, tear length, displacement status, chronicity, tissue quality, reducibility and associated chondral injury.

Vascular zone was classified according to the location of the tear relative to the peripheral meniscocapsular junction as red–red, red–white or white–white. Tears involving the peripheral vascularized third were classified as red–red, tears extending across the transition between vascularized peripheral and relatively avascular central meniscal tissue as red–white and tears confined predominantly to the central avascular portion as white–white. For prognostic analyses, red–red and red–white tears were grouped as vascular/peripheral tears and compared with white–white tears.

Tear chronicity was determined from the documented interval between the index injury and meniscal repair and was categorized as acute (<3 months) or chronic (≥3 months).

Tear morphology was classified into longitudinal vertical, bucket‐handle, radial, horizontal cleavage, complex, ramp and root tears [4, 18, 24]. Meniscal tissue quality was assessed intraoperatively according to structural integrity, macroscopic degenerative change and the ability of the tissue to securely retain sutures during fixation. Tissue was categorized as good when structural integrity was preserved without substantial macroscopic degeneration and secure suture purchase was achieved; intermediate when mild‐to‐moderate degeneration or reduced structural integrity was present but stable fixation remained achievable; and poor when substantial degeneration, friability or reduced suture‐holding capacity was documented.

Tissue quality was categorized as good when the meniscal tissue was structurally preserved without substantial macroscopic degeneration and demonstrated secure suture purchase; intermediate when mild‐to‐moderate degeneration or reduced tissue integrity was present but stable fixation remained achievable; and poor when substantial degeneration, friability or reduced suture‐holding capacity was documented. Articular cartilage status was graded using the Outerbridge classification.

Vascular zone was classified intraoperatively according to the location of the tear relative to the peripheral meniscocapsular junction as red–red, red–white or white–white and was extracted from the operative report using predefined criteria.

For prognostic analyses, red–red and red–white tears were grouped as vascular/peripheral tears, whereas white–white tears were categorized as avascular tears.

Procedure‐related variables included repair technique (all‐inside, inside‐out or hybrid), number of fixation sutures, calculated suture density, biological augmentation when performed and concomitant procedures. For clarity, ‘repair technique’ refers to the surgical approach used for fixation (all‐inside, inside‐out or hybrid), whereas ‘repair construct’ refers collectively to the fixation characteristics of the repair, including technique, number of sutures and suture density.

Biological augmentation consisted of marrow stimulation of the intercondylar notch to promote intra‐articular release of marrow‐derived blood and cellular elements. It was selectively performed at the discretion of the operating surgeon in tears considered to have reduced biological healing potential.

Suture density was defined as the number of fixation sutures divided by tear length and was analysed as a continuous variable.

### Outcome definition

The primary outcome was repair failure requiring subsequent ipsilateral meniscal reoperation. Routine postoperative MRI or second‐look arthroscopy was not required to define repair failure. Imaging findings without subsequent meniscal reoperation were therefore not classified as failures for the primary analysis.

Failure was defined as any repeat surgical intervention performed because of symptomatic recurrent meniscal pathology involving the previously repaired meniscus, including revision repair or partial or subtotal meniscectomy. Time to failure was calculated from the date of index meniscal repair to the date of the first subsequent meniscal reoperation. Patients without reoperation were censored at their most recent clinical follow‐up. Reoperation was selected as the primary endpoint because it provides an objective and clinically relevant measure of symptomatic repair failure and can be consistently identified in retrospective multicenter data. However, this definition does not capture asymptomatic structural failure or symptomatic failure managed nonoperatively [29, 34, 35].

### Statistical analysis

Continuous variables were assessed for normality using the Shapiro–Wilk test and inspection of histograms and *Q*–*Q* plots. Normally distributed variables are presented as mean ± standard deviation, whereas non‐normally distributed variables are reported as median with interquartile range (IQR). Categorical variables are expressed as frequencies and percentages.

Univariable comparisons were initially performed using the independent‐samples *t* test or Mann–Whitney *U* test for continuous variables and the *χ* ^2^ or Fisher exact test for categorical variables, as appropriate.

Repair survivorship was evaluated using Kaplan–Meier survival analysis, with differences between tear morphologies compared using the log‐rank test. Variables demonstrating clinical relevance or univariable association with failure were entered into multivariable Cox proportional hazards regression models to identify independent predictors of repair failure. The proportional hazards assumption was verified using Schoenfeld residuals before model construction. Hazard ratios are reported with corresponding 95% confidence intervals (CIs).

To determine the incremental prognostic contribution of tear morphology, two predefined multivariable models were developed. The reference model incorporated established patient‐, lesion‐ and procedure‐related predictors, whereas the extended model additionally included tear morphology. Incremental model performance was quantified using likelihood‐ratio testing, Harrell's concordance index, Akaike information criterion, calibration assessment and Brier score. Internal validation was performed using bootstrap resampling with 1000 repetitions to estimate optimism‐corrected model performance and calibration.

Prespecified sensitivity analyses were conducted by excluding isolated root tears, restricting analyses to isolated meniscal repairs without concomitant anterior cruciate ligament reconstruction and evaluating patients undergoing concomitant ligament reconstruction separately. Missing data were examined before analysis, and complete‐case analysis was performed when missingness was minimal; otherwise, multiple imputation using chained equations was considered when appropriate. Statistical significance was established at a two‐sided *p* value < 0.05. All analyses were performed using R statistical software (R Foundation for Statistical Computing).

## RESULTS

During the study period, 624 consecutive patients underwent arthroscopic meniscal repair and were screened for eligibility. After application of the predefined inclusion and exclusion criteria, 500 patients constituted the final study cohort (Figure 1). The most frequent reasons for exclusion were previous ipsilateral meniscal surgery (*n* = 38), revision meniscal repair (*n* = 24), incomplete operative documentation (*n* = 31), follow‐up shorter than 24 months (*n* = 21) and missing outcome data (*n* = 10).

The study cohort comprised 312 men (62.4%) and 188 women (37.6%), with a mean age of 28.9 ± 8.3 years (range, 14–52 years) and a mean BMI of 25.3 ± 3.4 kg/m^2^. Three hundred eighteen patients (63.6%) participated in pivoting sports before injury, including 174 (34.8%) competing at a competitive level. The median interval from injury to surgery was 4.2 months (IQR, 1.8–8.7 months).

Concomitant anterior cruciate ligament reconstruction was performed in 238 patients (47.6%), whereas 262 patients (52.4%) underwent isolated meniscal repair. The median clinical follow‐up was 45 months (IQR, 36–63 months), with complete follow‐up available for 482 patients (96.4%).

### Distribution of tear morphology

Longitudinal vertical tears represented the most common primary tear morphology, followed by bucket‐handle, radial, horizontal cleavage and complex tears (Tables 1, 2 and 2). Ramp and root involvement, when present, were recorded separately as anatomical tear characteristics rather than mutually exclusive primary morphology categories.

**Table 1:** Baseline characteristics of the study cohort (*n* = 500).

Variable	Value
Age, years	28.9 ± 8.3
Male sex	312 (62.4%)
Female sex	188 (37.6%)
Body mass index, kg/m^2^	25.3 ± 3.4
Current smoker	74 (14.8%)
Competitive athletes	226 (45.2%)
Pivoting sports participation	318 (63.6%)
Time from injury to surgery, months	5.8 ± 4.7
Concomitant ACL reconstruction	238 (47.6%)
Isolated meniscal repair	262 (52.4%)
Medial meniscus	286 (57.2%)
Lateral meniscus	214 (42.8%)
Follow‐up, months	45.2 ± 11.8
Repair failure (reoperation)	81 (16.2%)

Abbreviation: ACL, anterior cruciate ligament.

**Table 2:** Lesion characteristics according to tear morphology.

Characteristic	Longitudinal vertical (*n* = 168)	Bucket‐handle (*n* = 102)	Radial (*n* = 86)	Horizontal cleavage (*n* = 74)	Complex (*n* = 70)	*p* value
Medial meniscus, *n* (%)	88 (52.4)	74 (72.5)	48 (55.8)	40 (54.1)	36 (51.4)	**0.012**
Red–red/red–white vascular zone, *n* (%)	143 (85.1)	82 (80.4)	56 (65.1)	46 (62.2)	38 (54.3)	**<0.001**
Good tissue quality, *n* (%)	152 (90.5)	82 (80.4)	61 (70.9)	51 (68.9)	36 (51.4)	**<0.001**
Tear length, mm	18.6 ± 5.2	29.4 ± 8.5	11.3 ± 3.1	19.8 ± 6.7	24.6 ± 7.9	**<0.001**
Number of fixation sutures	3.8 ± 1.2	6.1 ± 1.7	2.8 ± 0.9	3.5 ± 1.0	5.0 ± 1.5	**<0.001**
Concomitant ACL reconstruction, *n* (%)	91 (54.2)	58 (56.9)	39 (45.3)	27 (36.5)	23 (32.9)	**0.018**
Repair failure, *n* (%)	22 (13.1)	20 (19.6)	16 (18.6)	11 (14.9)	12 (17.1)	0.410

*Note*: Bold values are statistically significant.

Abbreviation: ACL, anterior cruciate ligament.

Medial meniscal repairs accounted for 286 procedures (57.2%), whereas 214 repairs (42.8%) involved the lateral meniscus. Most lesions involved the posterior horn (61.8%), followed by the meniscal body (28.4%) and anterior horn (9.8%). No patient underwent concomitant repair of both the medial and lateral menisci at the index procedure.

Good tissue quality was documented in 289 repairs (57.8%), intermediate quality in 149 (29.8%) and poor tissue quality in 62 (12.4%). The median tear length was 22 mm (IQR, 16–31 mm). All‐inside repair was the most frequently performed technique (58.6%), followed by hybrid (24.2%) and inside‐out repair (17.2%). The median number of fixation sutures was 4 (IQR, 3–5), corresponding to a median suture density of 1.8 sutures/cm.

Importantly, biological characteristics were not evenly distributed across tear morphologies (Table 2). Good tissue quality was present in 90.5% of longitudinal tears but progressively less frequently in radial (70.9%), horizontal cleavage (68.9%) and complex tears (51.4%; *p* < 0.001). Similarly, involvement of the red–red or red–white vascular zones was more frequent in longitudinal tears (85.1%) than in radial (65.1%), horizontal cleavage (62.2%) and complex tears (54.3%; *p* < 0.001). Thus, tear morphologies traditionally associated with greater repair complexity also more frequently exhibited biological characteristics associated with repair failure.

### Repair failure

During follow‐up, 81 patients (16.2%) underwent subsequent ipsilateral meniscal reoperation and fulfilled the predefined definition of clinically consequential repair failure. Structural nonhealing identified exclusively by imaging was not included in this endpoint. The median time to failure was 17.6 months (IQR, 10.4–29.3 months).

Kaplan–Meier survivorship at 1, 2, 3 and 5 years was 96.8% (95% CI, 95.3–98.3%), 91.9% (95% CI, 89.4–94.4%), 87.8% (95% CI, 84.8–90.8%) and 82.6% (95% CI, 78.3–86.9%), respectively (Figure 2).

**Figure 2:**
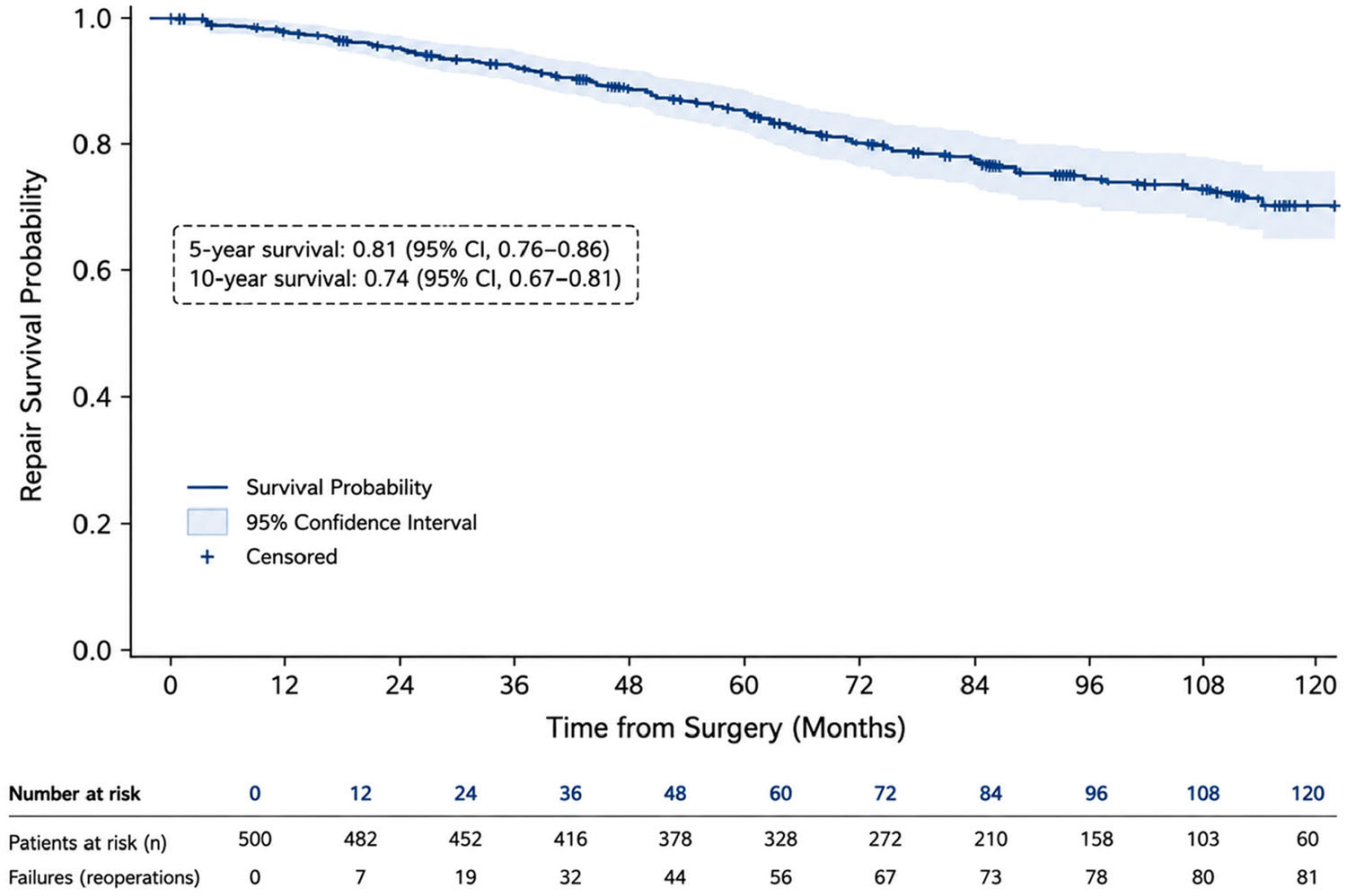
Kaplan–Meier survival analysis. Kaplan–Meier survival curve demonstrating repair survival following arthroscopic meniscal repair. Survival was defined as freedom from revision surgery. Censoring is indicated by tick marks, and shaded areas represent 95% CIs. Numbers at risk are displayed below the *x* axis. CIs, confidence intervals.

Five‐year survivorship according to tear morphology was 88.4% for longitudinal tears, 84.7% for bucket‐handle tears, 80.9% for radial tears, 86.3% for horizontal tears, 76.8% for complex tears, 89.1% for ramp lesions and 78.4% for root tears (log‐rank *p* = 0.012).

### Multivariable analysis

Variables entered into the multivariable Cox proportional hazards model included age, BMI, meniscal laterality, concomitant anterior cruciate ligament reconstruction, tear morphology, vascular zone, tissue quality, tear length, repair technique, suture density and cartilage status (Table 3; Figure 3).

**Table 3:** Multivariable Cox proportional hazards model for repair failure.

Variable	Hazard ratio	95% CI	*p* value
Medial meniscus	2.18	1.34–3.55	0.002
Poor tissue quality	2.74	1.69–4.43	<0.001
White–white vascular zone	1.89	1.16–3.08	0.011
Concomitant ACL reconstruction	0.63	0.40–0.98	0.041
Higher suture density (per additional suture/cm)	0.71	0.56–0.91	0.007
Age (per year)	1.01	0.98–1.03	0.49
BMI (per kg/m^2^)	1.02	0.97–1.08	0.39
Bucket‐handle tear	1.16	0.69–1.94	0.58
Radial tear	1.09	0.63–1.88	0.76
Horizontal tear	0.97	0.49–1.89	0.92
Complex tear	1.21	0.67–2.19	0.53

Abbreviations: ACL, anterior cruciate ligament; BMI, body mass index; CI, confidence interval.

**Figure 3:**
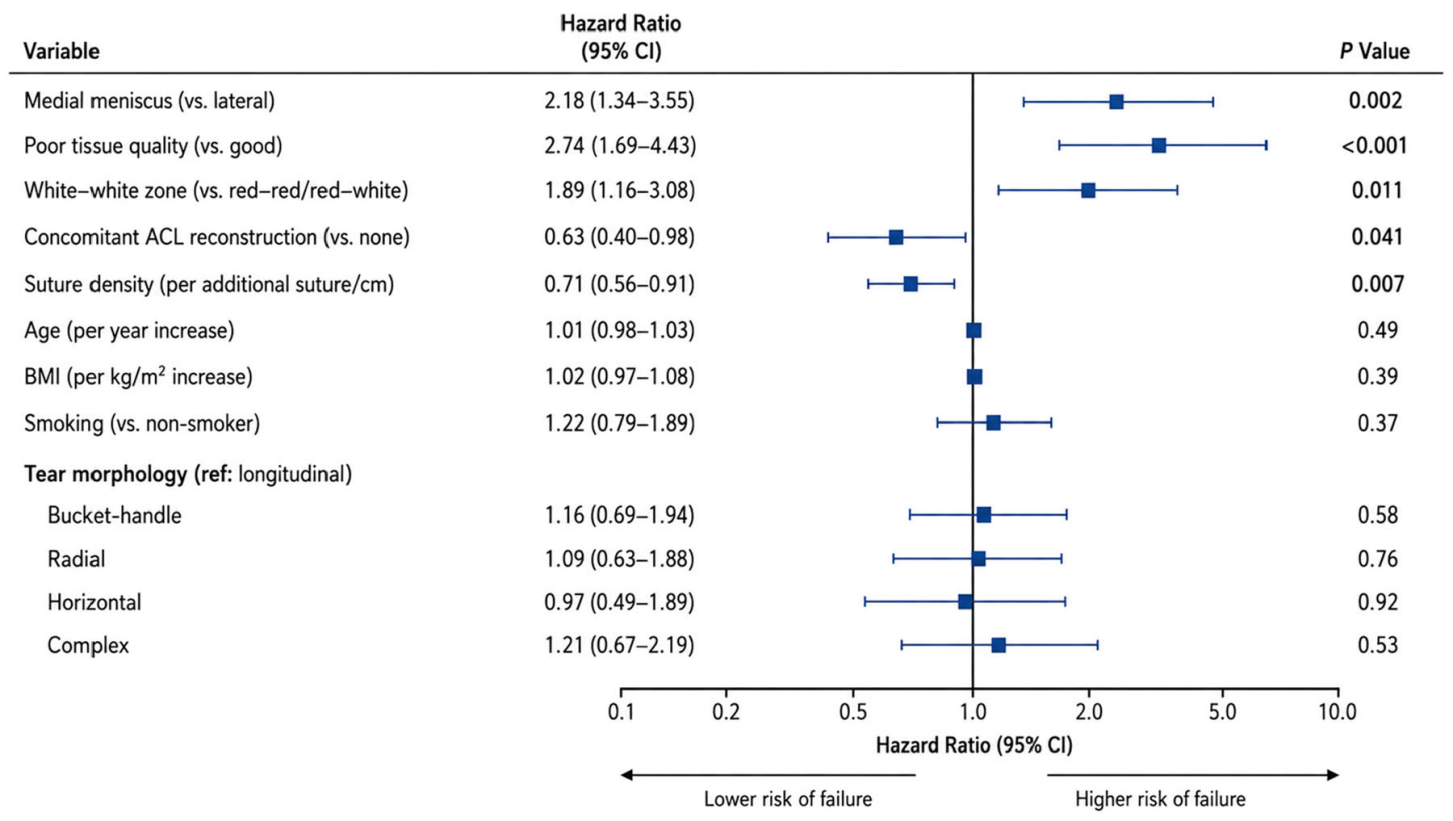
Multivariable predictors of repair failure. Forest plot showing adjusted hazard ratios derived from the multivariable Cox proportional hazards model. Hazard ratios are presented with 95% CIs. Variables with CIs crossing the line of unity were not independently associated with repair failure after adjustment. ACL, anterior cruciate ligament; BMI, body mass index; CI, confidence interval.

Independent predictors of repair failure included medial meniscal repair (hazard ratio [HR], 2.18; 95% CI, 1.36–3.49; *p* = 0.001), poor tissue quality (HR, 2.74; 95% CI, 1.61–4.67; *p* < 0.001), tears located within the avascular zone (HR, 1.89; 95% CI, 1.17–3.05; *p* = 0.009), lower suture density (HR per additional suture/cm, 0.71; 95% CI, 0.56–0.90; *p* = 0.004) and absence of concomitant anterior cruciate ligament reconstruction (HR, 1.67; 95% CI, 1.05–2.65; *p* = 0.031).

Compared with longitudinal tears, complex tears demonstrated an increased unadjusted risk of failure; however, after multivariable adjustment, tear morphology was no longer independently associated with repair failure. Hazard ratios for bucket‐handle, radial, horizontal, complex, ramp and root tears are presented in Table 4 and Figure 4.

**Table 4:** Incremental prognostic value of tear morphology.

Performance metric	Clinical model	Clinical model + tear morphology	Δ
Harrell C‐index	0.781	0.794	+0.013
Bootstrap‐corrected C‐index	0.774	0.786	+0.012
Akaike information criterion	648.7	643.9	−4.8
Likelihood ratio *χ* ^2^	58.3	66.7	+8.4
Likelihood ratio *p* value	—	0.038	—
Integrated Brier score	0.129	0.126	−0.003
Calibration slope	0.97	0.99	+0.02

**Figure 4:**
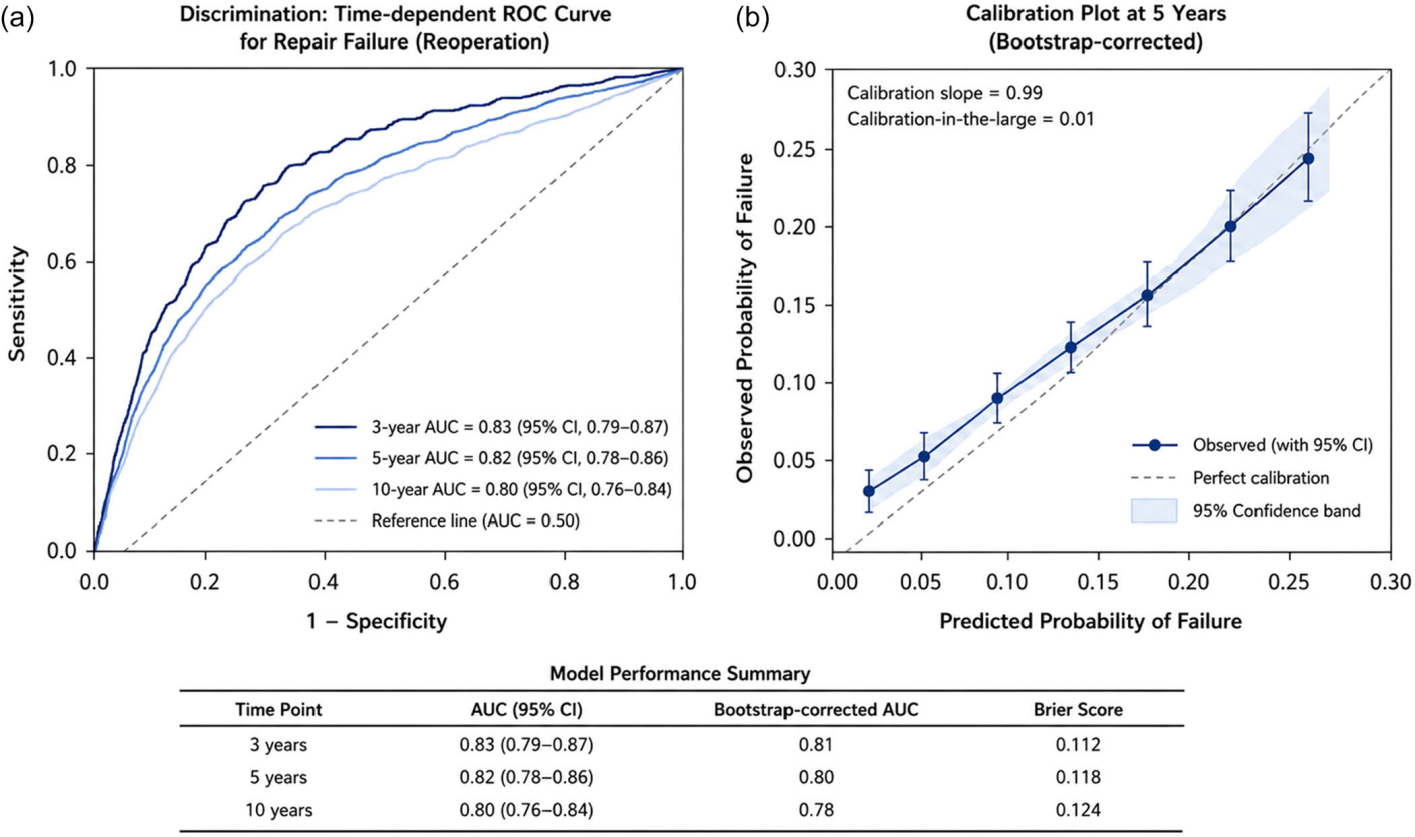
Performance of the multivariable prognostic model. (a) Time‐dependent ROC curve demonstrating model discrimination. (b) Calibration plot comparing predicted and observed probabilities of repair failure after internal bootstrap validation, illustrating good agreement across the spectrum of predicted risk. AUC, area under the receiver operating characteristic curve; CI, confidence interval; ROC, receiver operating characteristic.

### Incremental prognostic performance

The reference multivariable clinical model demonstrated good discrimination for repair failure (Harrell's C‐index, 0.782; optimism‐corrected C‐index, 0.769). Addition of tear morphology produced only a modest increase in model performance, with the C‐index improving to 0.794 and the optimism‐corrected C‐index to 0.778.

The likelihood‐ratio test favoured the extended model (*χ* ^2^ = 8.42, *p* = 0.038), although the increase in predictive discrimination remained limited. The optimism‐corrected Brier score improved from 0.128 to 0.124 following inclusion of tear morphology. Calibration plots demonstrated good agreement between predicted and observed probabilities throughout the spectrum of risk (Figure 5).

**Figure 5:**
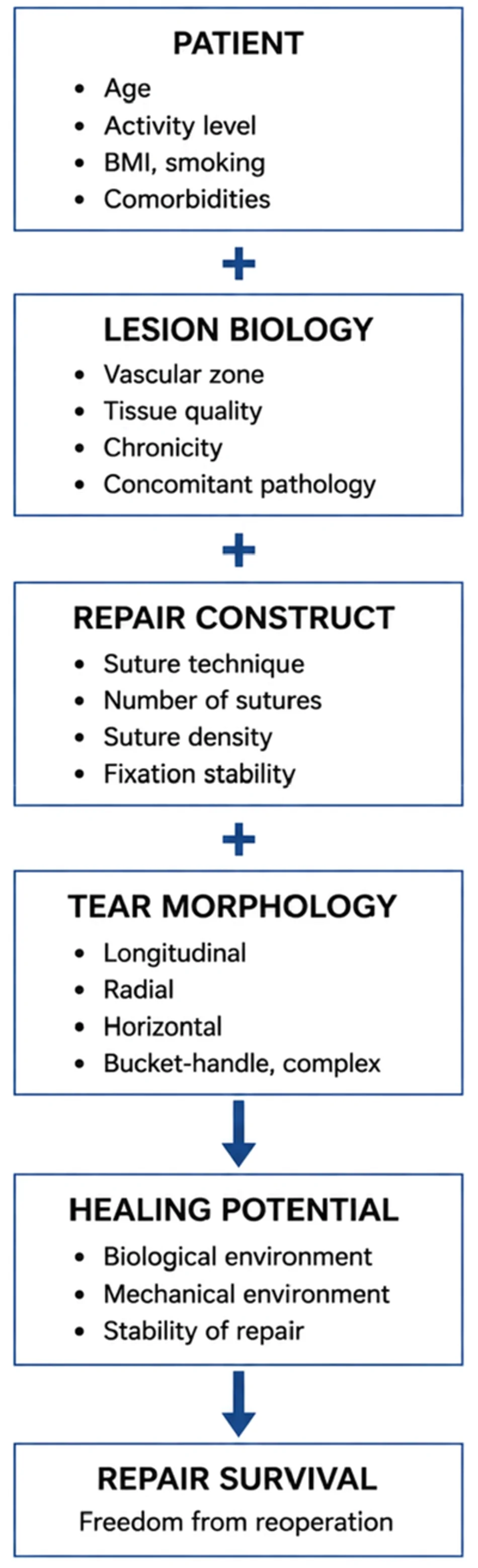
Contextual framework for prognostic assessment after meniscal repair. Conceptual framework illustrating the multidimensional determinants of meniscal repair prognosis. Rather than relying exclusively on tear morphology, healing potential emerges from the interaction between patient characteristics, lesion biology, repair construct and tear morphology. The combined biological and mechanical context ultimately determines repair survival. BMI, body mass index.

### Sensitivity analyses

Sensitivity analyses excluding isolated root tears, restricting the analysis to isolated meniscal repairs and separately evaluating repairs performed with concomitant anterior cruciate ligament reconstruction produced estimates comparable to those of the primary analysis. No clinically meaningful differences in the magnitude or direction of the observed associations were identified, and overall model discrimination and calibration remained unchanged.

## DISCUSSION

### Principal findings

The present study demonstrated that meniscal tear morphology alone provided limited independent prognostic information once patient‐, lesion‐ and procedure‐related variables were considered simultaneously. Although repair outcomes differed across tear configurations in unadjusted analyses, these associations were substantially attenuated after multivariable adjustment. Importantly, this finding should not be interpreted as indicating that tear morphology is clinically irrelevant. Rather, adverse biological characteristics were unequally distributed across morphologies: radial, horizontal cleavage and complex tears demonstrated lower frequencies of favourable vascular‐zone involvement and good tissue quality than longitudinal tears. These findings suggest that part of the apparent prognostic effect traditionally attributed to tear morphology may reflect the biological and mechanical characteristics that commonly accompany specific tear configurations. In contrast, contextual factors, including meniscal laterality, tissue quality, vascular zone involvement, repair construct and concomitant anterior cruciate ligament reconstruction, remained stronger determinants of repair survival [4, 11, 38]. The incremental contribution of tear morphology to overall model performance was modest, indicating that isolated morphologic classification incompletely captures the biological and mechanical complexity of meniscal healing. Collectively, these findings support a contextual prognostic approach rather than reliance on tear morphology alone when estimating the likelihood of successful meniscal preservation [3, 5, 14, 19, 23].

### Interpretation

The present findings are consistent with the biological complexity of meniscal healing. Importantly, tear morphology and the biological characteristics associated with repair failure were not evenly distributed across the cohort. Tear patterns traditionally considered more challenging to repair frequently exhibited less favourable biological characteristics. In the present cohort, good tissue quality was documented in 90.5% of longitudinal tears compared with 70.9% of radial, 68.9% of horizontal cleavage and 51.4% of complex tears (*p* < 0.001). Similarly, red–red or red–white vascular‐zone involvement was present in 85.1% of longitudinal tears but in only 65.1% of radial tears, 62.2% of horizontal cleavage and 54.3% of complex tears (*p* < 0.001). These findings are clinically relevant because poor tissue quality and avascular tear location were independently associated with repair failure in the multivariable analysis.

Accordingly, the attenuation of the association between tear morphology and failure after multivariable adjustment should not be interpreted as evidence that morphology is clinically unimportant. Rather, morphology may function partly as a marker of the biological and mechanical environment accompanying a given tear. Radial, horizontal cleavage, complex and root tears may more frequently coexist with adverse tissue characteristics, degenerative changes, altered vascularity or other factors that reduce healing potential, whereas simple longitudinal tears are more commonly encountered in a favourable traumatic and vascular environment [1, 12, 13, 25]. Thus, the higher failure rates historically attributed to certain tear configurations may be mediated, at least in part, by the characteristics that commonly accompany those morphologies rather than by tear geometry alone [22, 24, 37].

An important finding of the present study was that tear morphology and biological characteristics were not independent clinical phenomena. Longitudinal tears more frequently demonstrated good tissue quality and involvement of the vascularized peripheral meniscus, whereas radial, horizontal cleavage and complex tears more frequently exhibited less favourable tissue and vascular characteristics. These same characteristics were associated with repair failure in the adjusted analysis. Therefore, the attenuation of the association between tear morphology and failure after multivariable adjustment should not be interpreted as evidence that morphology is unimportant. Rather, morphology may function partly as a clinical marker of the underlying biological and mechanical environment in which healing occurs. In this context, the higher failure rates reported for certain complex tear patterns may be explained, at least in part, by the adverse tissue characteristics that commonly accompany those configurations rather than by tear geometry alone.

This interpretation is consistent with recent tear‐specific literature. Contemporary studies of radial, complex and posterior root repairs demonstrate clinically relevant failure rates, while also identifying patient‐, tissue‐, cartilage‐, rehabilitation‐ and lesion‐related characteristics that modify repair prognosis [1, 12, 13, 25]. Collectively, these observations suggest that morphology remains clinically informative but should be interpreted together with the biological characteristics that frequently accompany each tear pattern.

A similar relationship should be considered from a mechanical perspective. Restoration of circumferential hoop stress depends not only on tear configuration but also on tear location, residual functional meniscal tissue, fixation stability and load distribution across the repaired construct. A technically robust repair using appropriate suture configuration and adequate fixation may overcome the theoretical mechanical disadvantages of certain tear patterns, whereas suboptimal fixation may compromise healing even in tears traditionally considered favourable for preservation. Therefore, morphology should be regarded as only one component within a broader biomechanical environment [10, 18, 28, 36].

Recent tear‐specific studies similarly demonstrate clinically relevant failure rates after radial, complex and posterior root repair while identifying patient‐, tissue‐, cartilage‐, lesion‐ and rehabilitation‐related characteristics that modify prognosis [1, 12, 13, 25].

Vascularity provides an additional explanation for these observations. Tears extending into the peripheral vascularized zone are exposed to greater biological healing potential than lesions located predominantly within the avascular central meniscus [9, 10, 30]. Likewise, concomitant anterior cruciate ligament reconstruction may enhance the biological environment through marrow‐derived cellular recruitment and intra‐articular haematoma formation, partially compensating for unfavourable structural characteristics. Recent evidence regarding biological augmentation further supports the concept that optimization of the biological environment may influence healing independently of tear morphology itself [21, 23].

Finally, selection bias inherent to contemporary meniscal preservation should be acknowledged. Surgeons rarely attempt repair indiscriminately. Instead, treatment decisions already integrate tissue quality, vascularity, reducibility, chronicity and technical feasibility before repair is undertaken. Consequently, repair cohorts represent a biologically selected population in which the independent prognostic effect of morphology is expected to be less pronounced than in an unselected spectrum of meniscal injuries.

### Comparison with previous literature

The overall repair survival observed in the present cohort was consistent with contemporary literature. Five‐year survivorship was 82.6%, corresponding to a cumulative failure rate of approximately 17%, which closely parallels systematic reviews and meta‐analyses reporting failure or revision rates of approximately 19%–20% at mid‐ to long‐term follow‐up [26, 27, 29, 34, 35]. This concordance supports the external clinical plausibility of the present cohort despite differences among studies in tear configuration, concomitant ligament reconstruction, repair technique and failure definition.

The present findings align with recent evidence suggesting that tear morphology should not be interpreted as an isolated predictor of repair failure. Contemporary studies have demonstrated that medial meniscal repairs consistently exhibit higher failure rates than lateral repairs, while the independent contribution of tear morphology becomes less consistent after adjustment for additional prognostic variables [7, 8]. Similar conclusions emerge from large meta‐analyses, which identified considerable heterogeneity in repair survival attributable to patient selection, associated ligament reconstruction, repair technique, follow‐up duration and failure definitions rather than morphology alone [32, 34, 38, 39].

Studies focused on bucket‐handle tears have shown that satisfactory clinical outcomes can be achieved despite relatively high structural failure rates, emphasizing that preservation remains preferable whenever technically feasible [2, 3, 4, 5, 14, 19, 23, 37]. Likewise, systematic reviews evaluating radial and horizontal tears have demonstrated that successful repair is achievable across multiple tear configurations provided appropriate surgical techniques and patient selection are applied [18, 22, 24, 28, 36]. More recent evidence regarding isolated medial meniscal repair further reinforces the importance of biological and anatomical context over morphology alone when estimating repair prognosis [10]. Overall, the present findings complement rather than contradict previous tear‐specific literature. Differences in failure rates among tear configurations remain clinically relevant; however, the present analysis suggests that these differences should not necessarily be attributed to tear geometry itself. Tear patterns associated with higher reported failure rates also tend to occur in different biological and mechanical environments, including differences in tissue quality, vascularity, chronicity, chondral status and fixation requirements [1, 12, 13, 25]. The limited incremental prognostic value observed after adjustment therefore suggests that morphology captures part of this underlying risk context but provides relatively little additional prognostic information once these characteristics are considered directly [6, 9, 30, 33].

### Clinical implications

The present findings suggest that surgical decision‐making should extend beyond descriptive tear classification. Bucket‐handle, radial, horizontal cleavage, complex or root morphology should remain an important component of surgical assessment but should not independently determine whether a meniscal tear is considered repairable or whether failure is anticipated. Instead, morphology should be interpreted together with the tissue and mechanical characteristics that commonly accompany each tear pattern, including tissue quality, vascular zone involvement, chronicity, meniscal laterality, chondral status, concomitant anterior cruciate ligament reconstruction and fixation requirements. Instead, treatment decisions should integrate tissue quality, vascular zone involvement, meniscal laterality, chronicity, concomitant anterior cruciate ligament status, fixation strategy and overall biological healing potential [16]. This individualized approach is consistent with contemporary evidence demonstrating favourable long‐term outcomes after appropriately selected meniscal repair across different age groups and tear patterns while avoiding unnecessary meniscectomy [6, 9, 26, 27, 30, 33]. Accordingly, prognostic assessment should evolve from isolated morphologic descriptors toward comprehensive contextual evaluation capable of supporting individualized surgical planning and patient counselling [15, 17, 20, 31].

### Strengths and limitations

This study possesses several notable strengths. It included a large cohort of 500 consecutive arthroscopic meniscal repairs managed using contemporary preservation principles, thereby reducing selection bias associated with highly selected series. Time‐dependent outcomes were appropriately analysed using Kaplan–Meier survival analysis and multivariable Cox proportional hazards modelling, while internal bootstrap validation provided a robust assessment of model stability and minimized optimism in predictive performance estimates. Furthermore, the simultaneous evaluation of patient‐, lesion‐ and procedure‐related variables allowed estimation of the incremental prognostic contribution of tear morphology within a comprehensive clinical framework.

Several limitations should also be acknowledged. The retrospective observational design remains susceptible to residual confounding despite multivariable adjustment. External validation was not available, and the proposed prognostic model should therefore be confirmed in independent cohorts before routine clinical implementation.

In addition, tissue quality was based on intraoperative macroscopic assessment and was not subjected to independent blinded or interobserver reliability testing. Although predefined clinical characteristics were used to categorize tissue quality, this assessment remains partly subjective and may introduce observer‐dependent misclassification.

Finally, repair failure was defined by subsequent meniscal reoperation rather than systematic postoperative MRI or second‐look arthroscopy. Consequently, asymptomatic structural failures, persistent or recurrent tears identified on imaging but managed nonoperatively, and symptomatic patients who elected not to undergo further surgery may have been classified as nonfailures. The reported failure rate should therefore be interpreted as the rate of clinically consequential failure resulting in reoperation rather than the prevalence of structural nonhealing.

## CONCLUSION

Meniscal tear morphology was associated with repair survival in unadjusted analysis but was not independently associated with repair failure after adjustment for patient‐, meniscal‐ and repair‐related characteristics.

## AUTHOR CONTRIBUTIONS

**Francisco Endara Urresta**: Conceptualization; methodology; supervision; writing—review and editing. **Horacio Rivarola, Cristian Collazo, Marcos Palanconi** and **Marcos Meninato**: Conceptualization; methodology; validation; writing—review and editing. **Alejandro Barros Castro** and **Carlos Peñaherrera‐Carrill**: Investigation; data curation; writing—review and editing. All authors have read and approved the final version of the manuscript and agree to be accountable for all aspects of the work.

## FUNDING INFORMATION

The authors have no funding to report.

## CONFLICT OF INTEREST STATEMENT

The authors declare no conflicts of interest.

## ETHICS STATEMENT

This study was approved by Clinica Arthros (ARTC‐0074).

## Data Availability

The de‐identified data that support the findings of this study are available from the corresponding author upon reasonable request. Individual‐level clinical data that could potentially permit patient identification are not publicly available because of ethical and privacy restrictions.

## References

[1] Agonias K , Knell G , Pacicca D , Heyworth BE , Wilson P , Shea K , et al. Isolated meniscus repairs in youth have a 19% complication rate and 9% failure rate with variations by tear type, location, range of motion protocol, and sex at 8 months. Arthroscopy. 2026;42(10):2161–2172. 10.1002/arj.70361 42443128

[2] Alhamdi H , Seil R , Hoffmann A , et al. High failure rate after medial meniscus bucket‐handle tear repair in the stable knee. Orthop Traumatol Surg Res. 2024;110(2):103737. 10.1016/j.otsr.2023.103737 37898297

[3] Ardizzone CA , Houck DA , McCartney DW , Vidal AF , Frank RM . All‐inside repair of bucket‐handle meniscal tears: clinical outcomes and prognostic factors. Am J Sports Med. 2020;48(2):3386–3393. 10.1177/0363546520906141 32195594

[4] Costa GG , Grassi A , Zocco G , Graceffa A , Lauria M , Fanzone G , et al. What is the failure rate after arthroscopic repair of bucket‐handle meniscal tears? A systematic review and meta‐analysis. Am J Sports Med. 2022;50(6):1742–1752. 10.1177/03635465211015425 34161741

[5] El Helou A , Ezzeddine J , Daher M , et al. Failure rates of repaired bucket‐handle tears of the medial meniscus concomitant with anterior cruciate ligament reconstruction: a cohort study. Am J Sports Med. 2023;51(4):949–956. 10.1177/03635465221148497 36734511

[6] Engler ID , Moradian JR , Pockros BM , Schirmeister CM , Richmond JC , Salzler MJ . Patient‐reported outcomes of meniscal repair and meniscectomy in patients 40 years of age and older show similar good results. Knee Surg Sports Traumatol Arthrosc. 2021;29(9):2911–2917. 10.1007/s00167-020-06299-5 33025055

[7] von Essen C , Cristiani R , Kekki C , Rizvanovic D , Stålman A . Isolated meniscal repair, medial meniscus repair and older age increase failure risk after all‐inside meniscal repair: a cohort study of 2264 patients. Knee Surg Sports Traumatol Arthrosc. 2026;34(6):1977–1985. 10.1002/ksa.12780 PMC13266934 40622005

[8] von Essen C , Cristiani R , Rizvanovic D , Stålman A . Impact of tear morphology and meniscal laterality on repair failure in stable knees: medial repairs show over twofold risk. Knee Surg Sports Traumatol Arthrosc. 2025. 10.1002/ksa.70166 41172977

[9] Everhart JS , Higgins JD , Poland SG , Abouljoud MM , Flanigan DC . Meniscal repair in patients age 40 years and older: a systematic review of 11 studies and 148 patients. Knee. 2018;25(6):1142–1150. 10.1016/j.knee.2018.09.009 30414793

[10] Farinelli L , Meena A , Montini D , Patralekh MK , Piritore G , Grassi M , et al. Failure rate of isolated medial meniscus repair in the stable knee: systematic review and meta‐analysis. Knee Surg Sports Traumatol Arthrosc. 2025;33(4):1333–1344. 10.1002/ksa.12441 39189111

[11] Hagmeijer MH , Kennedy NI , Tagliero AJ , Levy BA , Stuart MJ , Saris DBF , et al. Long‐term results after repair of isolated meniscal tears among patients aged 18 years and younger: an 18‐year follow‐up study. Am J Sports Med. 2019;47(4):799–806. 10.1177/0363546519826088 30802135

[12] Helito CP , da Silva AGM , Guimarães TM , Jacinto MRR , Sobrado MF , Gobbi RG . Female sex, high body mass index, more pronounced femoral chondral injury, and poor rehabilitation compliance were associated with medial meniscus posterior root tears repair failure. Arthroscopy. 2026;42(1):233–243. 10.1002/arj.70019 41838540

[13] Helito CP , Moreira da Silva AG , Familiari F , Simonetta R , Andrade MC , Gomes DE , et al. Clinical outcomes of repaired radial meniscal tears: a retrospective study with minimum 2‐year follow‐up. Knee. 2025;53:285–292. 10.1016/j.knee.2025.01.009 39922177

[14] Kalifis G , Raoulis V , Panteliadou F , Liantsis A , D'Ambrosi R , Hantes M . Long‐term follow‐up of bucket‐handle meniscal repairs: chondroprotective effect outweighs high failure risk. Knee Surg Sports Traumatol Arthrosc. 2022;30(7):2209–2214. 10.1007/s00167-021-06787-2 34741626

[15] Kang DG , Park YJ , Yu JH , Oh JB , Lee DY . A systematic review and meta‐analysis of arthroscopic meniscus repair in young patients: comparison of all‐inside and inside‐out suture techniques. Knee Surg Relat Res. 2019;31(1):1–11. 10.5792/ksrr.17.078 PMC6425899 30497231

[16] Keller RE , O'Donnell EA , Medina GIS , Linderman SE , Cheng TTW , Sabbag OD , et al. Biological augmentation of meniscal repair: a systematic review. Knee Surg Sports Traumatol Arthrosc. 2022;30(6):1915–1926. 10.1007/s00167-021-06849-5 35258647

[17] Krych AJ , Reardon P , Sousa P , Levy BA , Dahm DL , Stuart MJ , et al. Clinical outcomes after revision meniscus repair. Arthroscopy. 2016;32(9):1831–1837. 10.1016/j.arthro.2016.01.070 27132771

[18] Kurzweil PR , Lynch NM , Coleman S , Kearney B . Repair of horizontal meniscus tears: a systematic review. Arthroscopy. 2014;30(11):1513–1519. 10.1016/j.arthro.2014.05.038 25108905

[19] Lamba A , Hevesi M , Pan X , Boos AM , Wang AS , Levy BA , et al. Long‐term comparative outcomes of all‐inside versus inside‐out repair of bucket‐handle meniscal tears: a cohort study. Orthop J Sports Med. 2024;12(12):23259671241296899. 10.1177/23259671241296899 PMC11662328 39711605

[20] Lee GP , Diduch DR . Deteriorating outcomes after meniscal repair using the meniscus arrow in knees undergoing concurrent anterior cruciate ligament reconstruction: increased failure rate with long‐term follow‐up. Am J Sports Med. 2005;33(8):1138–1141. 10.1177/0363546505275348 16000655

[21] Li Z , Weng X . Platelet‐rich plasma use in meniscus repair treatment: a systematic review and meta‐analysis of clinical studies. J Orthop Surg Res. 2022;17(1):446. 10.1186/s13018-022-03293-0 PMC9548158 36209223

[22] Milliron EM , Magnussen RA , Cavendish PA , Quinn JP , DiBartola AC , Flanigan DC . Repair of radial meniscus tears results in improved patient‐reported outcome scores: a systematic review. Arthrosc Sports Med Rehabil. 2021;3(3):e967–e980. 10.1016/j.asmr.2021.03.002 PMC8220631 34195666

[23] Moatshe G , Cinque ME , Godin JA , Vap AR , Chahla J , LaPrade RF . Comparable outcomes after bucket‐handle meniscal repair and vertical meniscal repair can be achieved at a minimum 2 years' follow‐up. Am J Sports Med. 2017;45(13):3104–3110. 10.1177/0363546517719244 28806092

[24] Moulton SG , Bhatia S , Civitarese DM , Frank RM , Dean CS , LaPrade RF . Surgical techniques and outcomes of repairing meniscal radial tears: a systematic review. Arthroscopy. 2016;32(9):1919–1925. 10.1016/j.arthro.2016.03.029 27234653

[25] Myers RJ , Agner KE , Huntley KS , Rock CL , Milliron EM , Cavendish PA , et al. Midterm outcomes following repair of complex meniscus tears: a retrospective analysis of failure risk and postoperative complications. J Knee Surg. 2026. 10.1055/a-2865-3315 42242240

[26] Nepple JJ , Block AM , Eisenberg MT , Palumbo NE , Wright RW . Meniscal repair outcomes at greater than 5 years: a systematic review and meta‐analysis. J Bone Jt Surg. 2022;104(14):1311–1320. 10.2106/JBJS.21.01303 35856932

[27] Nepple JJ , Dunn WR , Wright RW . Meniscal repair outcomes at greater than five years: a systematic literature review and meta‐analysis. J Bone Jt Surg Am. 2012;94(24):2222–2227. 10.2106/JBJS.K.01584 PMC3528023 23318612

[28] Oosten J , Yoder R , DiBartola A , Bowler J , Sparks A , Duerr R , et al. Several techniques exist with favorable biomechanical outcomes in radial meniscus tear repair: a systematic review. Arthroscopy. 2022;38(8):2557–2578.e1. 10.1016/j.arthro.2022.02.010 35189305

[29] Ow ZGW , Law MSN , Ng CH , Krych AJ , Saris DBF , Debieux P , et al. All‐cause failure rates increase with time following meniscal repair despite favorable outcomes: a systematic review and meta‐analysis. Arthroscopy. 2021;37(12):3518–3528. 10.1016/j.arthro.2021.05.033 34058318

[30] Poland S , Everhart JS , Kim W , Axcell K , Magnussen RA , Flanigan DC . Age of 40 years or older does not affect meniscal repair failure risk at 5 years. Arthroscopy. 2019;35(5):1527–1532. 10.1016/j.arthro.2018.11.061 31000396

[31] Pujol N , Tardy N , Boisrenoult P , Beaufils P . Long‐term outcomes of all‐inside meniscal repair. Knee Surg Sports Traumatol Arthrosc. 2015;23(1):219–224. 10.1007/s00167-013-2553-5 23740324

[32] Rönnblad E , Barenius B , Eriksson K . Predictive factors for failure of meniscal repair: a retrospective dual‐center analysis of 918 consecutive cases. Orthop J Sports Med. 2020;8(3):2325967120905529. 10.1177/2325967120905529 PMC7137129 32284936

[33] Rothermel SD , Smuin D , Dhawan A . Are outcomes after meniscal repair age dependent? A systematic review. Arthroscopy. 2018;34(3):979–987. 10.1016/j.arthro.2017.08.287 29273257

[34] Schweizer C , Hanreich C , Tscholl PM , Blatter S , Windhager R , Waldstein W . Meniscal repair outcome in 3829 patients with a minimum follow‐up from 2 years up to 5 years: a meta‐analysis on the overall failure rate and factors influencing failure. Am J Sports Med. 2024;52(3):822–831. 10.1177/03635465231158385 37022676

[35] Schweizer C , Hanreich C , Tscholl PM , Ristl R , Apprich S , Windhager R , et al. Nineteen percent of meniscus repairs are being revised and failures frequently occur after the second postoperative year: a systematic review and meta‐analysis with a minimum follow‐up of 5 years. Knee Surg Sports Traumatol Arthrosc. 2022;30(7):2267–2276. 10.1007/s00167-021-06770-x PMC9206598 34671817

[36] Shanmugaraj A , Tejpal T , Ekhtiari S , Gohal C , Horner N , Hanson B , et al. The repair of horizontal cleavage tears yields higher complication rates compared to meniscectomy: a systematic review. Knee Surg Sports Traumatol Arthrosc. 2020;28(3):915–925. 10.1007/s00167-019-05557-5 31187178

[37] Thaunat M , Fournier G , O'Loughlin P , Kouevidjin BT , Clowez G , Borella M , et al. Clinical outcome and failure analysis of medial meniscus bucket‐handle tear repair: a series of 96 patients with a minimum 2‐year follow‐up. Arch Orthop Trauma Surg. 2020;140(11):1649–1654. 10.1007/s00402-020-03346-1 31993744

[38] Turcotte JJ , Maley AD , Levermore SB , Petre BM , Redziniak DE . Risk factors for all‐inside meniscal repair failure in isolation and in conjunction with anterior cruciate ligament reconstruction. Knee. 2021;28:9–16. 10.1016/j.knee.2020.10.012 33278740

[39] Yeo DYT , Suhaimi F , Parker DA . Factors predicting failure rates and patient‐reported outcome measures after arthroscopic meniscal repair. Arthroscopy. 2019;35(11):3146–3164.e2. 10.1016/j.arthro.2019.06.016 31699269

